# Delayed Onset Minimal Change Disease as a Manifestation of Lupus Podocytopathy

**DOI:** 10.3390/clinpract11040089

**Published:** 2021-10-06

**Authors:** Rasha Aly, Xu Zeng, Ratna Acharya, Kiran Upadhyay

**Affiliations:** 1 Department of Pediatrics, Division of Pediatric Nephrology, University of Florida, Gainesville, FL 32610, USA; rashaaly@ufl.edu; 2 Department of Pathology, Division of Anatomic Pathology, University of Florida, Gainesville, FL 32610, USA; xu.zeng@ufl.edu; 3 Department of Pediatrics, Division of General Pediatrics, University of Florida, Gainesville, FL 32610, USA; racharya@ufl.edu

**Keywords:** SLE, nephritis, podocytopathy, minimal change disease

## Abstract

Lupus podocytopathy (LP) is an uncommon manifestation of systemic lupus erythematosus (SLE) and is not included in the classification of lupus nephritis. The diagnosis of LP is confirmed by the presence of diffuse foot process effacement in the absence of capillary wall deposits with or without mesangial immune deposits in a patient with SLE. Here we describe a 13-year-old female who presented with nephrotic syndrome (NS) seven years after the diagnosis of SLE. The renal function had been stable for seven years since the SLE diagnosis, as manifested by the normal serum creatinine, serum albumin and absence of proteinuria. Renal biopsy showed evidence of minimal change disease without immune complex deposits or features of membranous nephropathy. Renal function was normal. The patient had an excellent response to steroid therapy with remission within two weeks. The patient remained in remission five months later during the most recent follow-up. This report highlights the importance of renal histology to determine the accurate etiology of NS in patients with SLE. Circulating factors, including cytokines such as interleukin 13, may play a role in the pathophysiology of LP and needs to be studied further in future larger studies.

## 1. Introduction

Systemic lupus erythematosus (SLE) is an inflammatory autoimmune disease affecting multiple-organ systems with periods of relapses and remissions [1,2]. About 15 to 20% of all SLE patients have a disease onset before the age of 16 years and is termed pediatric SLE (p-SLE). Renal involvement, also called lupus nephritis (LN), is common in SLE, with 60% of cases presenting with early LN at the time of diagnosis of SLE [3,4]. The clinical manifestations of LN include abnormal renal function, hematuria, proteinuria and elevated blood pressures, among others. Common renal biopsy findings in LN are endocapillary proliferation, mesangial hypercellularity, full-house immunofluorescence and various electron dense deposits in the mesangium, subepithelial or subendothelial regions. LN is a major risk factor for mortality and morbidity in patients with SLE and a significant portion of these patients go on to develop end stage renal disease (ESRD) [5].

Nephrotic syndrome (NS) is manifested by nephrotic-range proteinuria, hypoalbuminemia and edema. Effacement of the podocyte foot processes is the hallmark of NS. Steroids and other immunosuppressive agents are the mainstay of treatment, but patients may or may not respond to these therapies. Although most cases are idiopathic, NS has been described in patients with SLE, such as membranous nephropathy in class V LN [6]. Minimal change disease (MCD) and focal segmental glomerulosclerosis (FSGS) can also be rarely associated with SLE [7,8,9,10].

Clinically, proteinuria and hematuria are characteristic features in patients with LN and have traditionally been thought to be the result of immune complex deposition and endocapillary proliferation causing a disruption to the glomerular filtration barrier. However, in a subset of nephrotic lupus patients, there is no evidence of the endocapillary proliferation, necrosis or crescents, along with absence of subepithelial or subendothelial deposits. Instead, if the deposits are present, they are confined to the mesangium, and there is an extensive podocyte effacement, leading to nephrotic-range proteinuria, and is termed lupus podocytopathy (LP) [10,11,12,13]. The aim of this study is to report late onset LP as one of the under-recognized entities in patients with SLE who do not have overt LN. Here, we describe a 13-year-old female who presented with delayed onset MCD as a manifestation of LP seven years after the diagnosis of SLE without prior evidence of LN. This was a retrospective chart review case study.

## 2. Case Report

A 13-year-old African American (AA) female presented with decreased urine output, puffiness of eyes and face, pitting edema of lower extremities and new onset shortness of breath for 4 days. There was no history of recent sore throat, abdominal pain, fever, usage of new medication including nonsteroidal anti-inflammatory agents or family or school stressors. The patient was diagnosed with SLE/autoimmune overlap syndrome with arthritis at the age of six years. Initial presentation at that time was with pericardial and pleural effusions (requiring a pericardial drain and chest tube placement), elevated anti-nuclear (ANA), anti-smith, anti-double-stranded (ds) DNA, ribonucleoprotein and SSA antibodies, Coombs positive autoimmune hemolytic anemia, and hypocomplementemia. The renal function, blood pressure and urinalysis were normal with no proteinuria or hematuria, indicating no renal involvement and hence the patient did not undergo renal biopsy initially. Initial management included intravenous pulse steroid and Rituximab therapy. The overlap syndrome remained in remission with every four weeks etanercept infusion.

Past medical history was significant for Triple X syndrome (Trisomy X), petit mal seizures, autism, attention deficit hyperactivity disorder, early puberty and steroid induced hypertension. Family history was not suggestive of lupus, NS, other autoimmune diseases, renal failure, dialysis or kidney transplantation.

On examination, vital signs showed an afebrile child with heart rate of 90 beats per minute, respiratory rate 16 per minute, blood pressure 150/110 mm Hg, and oxygen saturation of 98% on room air. Physical examination showed puffiness of eyes and face, decreased breath sounds bilaterally, basal rales and rhonchi, and 1+ bilateral pitting edema in the legs. There were no malar or discoid rash, oral ulcers or photosensitivity. Mental status was normal without seizures. There was no arthritis.

### 2.1. Results

Laboratory investigations revealed nephrotic-range proteinuria with spot urine protein creatinine ratio of 11 mg/mg (normal < 0.2 mg/mg), hypoalbuminemia (serum albumin 1.7 gm/dL), and serum creatinine of 0.8 mg/dL with a Schwartz estimated glomerular filtration rate of 84 mL/min/1.73 m^2^. Serum electrolytes showed mild hyperkalemia (5.4 meq/L) and metabolic acidosis (serum bicarbonate 17 meq/L), positive ANA with a titer 1:1280 in a nuclear speckled pattern, positive dsDNA and anti-smith antibodies, and normal serum complements.

**Figure 1:**
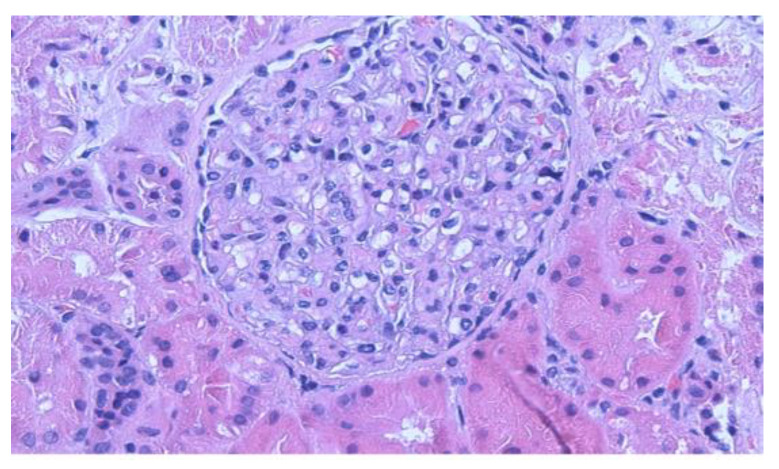
Light microscopy of kidney biopsy demonstrates normal mesangial cellularity (H&E 200×).

**Figure 2:**
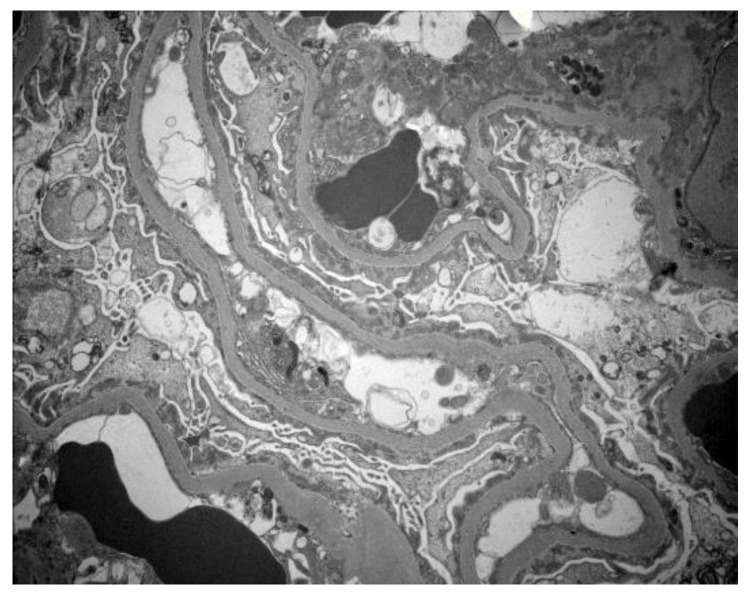
Electron microscopy demonstrates diffuse, almost complete podocyte foot processes effacement without immune mesangial or capillary wall deposits (Direct magnification 20,000×).

Chest X-ray revealed bilateral pleural effusions, right larger than left. Echocardiogram showed no pericardial effusion and evidence of mild left ventricular hypertrophy. Renal sonogram demonstrated a slight increase in renal echogenicity but without hydronephrosis. A percutaneous renal biopsy was obtained a week after the onset of current signs and symptoms. Biopsy showed three portions of renal cortical and cortico-medullary tissue with 22 glomeruli and three blood vessels. Six glomeruli were available for immunofluorescence and 1 glomerulus was selected for electron microscopy. Light microscopy showed normal appearing glomerular capillary loops, mesangium, tubulointerstitium, and blood vessels (Figure 1). There was no glomerular segmental sclerosis. Immunofluorescence study was negative for IgA, IgG, IgM, C3, C1q, Kappa, and lambda light chains. Electron microscopy showed diffuse, foot process effacement with no electron dense deposits in the mesangium and capillary walls, indicating severe podocyte injury (Figure 2). These findings were most consistent with MCD with no features of membranous LN.

### 2.2. Management

The patient was treated with albumin/diuretic infusions for the symptomatic relief of edema and a course of intravenous methylprednisolone 10 mg/kg daily for three days. Later, the patient was transitioned to oral steroid 60 mg/m^2^/day for six weeks, followed by 40 mg/m^2^ every other day for another six weeks. Home medications included amlodipine, enalapril, hydroxychloroquine, pantoprazole, and etanercept infusion every 4 weeks as a maintenance agent for the history of arthritis.

### 2.3. Outcome

The edema resolved within two weeks. At two weeks follow-up, the urinalysis showed only trace proteinuria. The patient completed 12 weeks course of oral steroid and a follow-up spot urine protein creatinine ratio after 12 weeks was 0.1 mg/mg. Serum albumin had normalized. Renal function remained stable. Blood pressures were stable on amlodipine and enalapril. During the most recent follow-up after five months, the patient has not had relapse of the MCD.

## 3. Discussion

LN is common in patients with SLE. Most of the studies show that the risk of LN is highest in the initial five year following SLE diagnosis [5]. However, 31–48% of patients who do not have LN at the time of diagnosis develop LN at some point in their disease course, up to 19 years later [5,14,15,16,17,18]. Our patient had not shown manifestations of LN, instead the presentation was with extremely rare manifestation of MCD seven years after the diagnosis of SLE. Nishihara et al. described a new onset lupus in a 17-year-old female with MCD in remission [19]. Similarly, MCD has also been described in adults with SLE. One study described an adult female with steroid sensitive MCD associated with SLE followed by a relapse 15 months later with a concomitant reactivation of SLE and second biopsy showing class II LN [20]. Dube et al. presented seven patients with SLE and MCD [7]. Hence, MCD is an under-recognized entity in SLE.

In SLE, nephrotic-range proteinuria and NS typically signifies the presence of a proliferative LN (class III/IV) and/or membranous LN (class V, with or without concomitant class III or IV lesions). However, in rare instances, SLE patients with NS can have renal biopsy findings of normal appearing glomeruli, with or without mesangial proliferation, on light microscopy; the absence of subepithelial or subendothelial deposits on immunofluorescence and electron microscopy; and diffuse foot process effacement on electron microscopy. This pattern, termed LP, represents about 1% of LN biopsies [21,22]; however, one report found that about 8% of patients with pediatric LN may have LP [23].

Various morphologies of LP have been described. In one of the largest studies, Hu et al. described LP in 50 out of 3750 (1.3%) cases; 13 had MCD, 28 had mesangial proliferation and 9 had FSGS [21]. A total of 68% had low C3. All had extensive foot process effacement and had clinical NS. A total of 34% had AKI, more in the FSGS subgroup. A total of 94% achieved remission with immunosuppressive therapy after a median time of four weeks. These patients had a SLE diagnosis duration of median time 4–10 months. None of them progressed to ESRD. In our report, MCD as a manifestation of LP occurred seven years after the diagnosis of SLE.

The exact pathophysiology of LP is unclear. In the absence of immune deposits, the possible pathogenesis could be similar to that of idiopathic MCD. T and B cell dysfunction along with circulating immune factors that alter the glomerular permeability leading to nephrotic-range proteinuria have been proposed as possible mechanisms of podocytopathy in idiopathic MCD. One study examined the mRNA expression of interleukin-2 (IL-2), interferon-gamma, IL-4, and IL-13 from CD4^+^ and CD8^+^ T-cells in 55 children with steroid-responsive nephrotic patients in relapse and remission and demonstrated increased CD4^+^ and CD8^+^ IL-13 mRNA expression in patients with nephrotic relapse as compared to remission, normal, and patient controls [24]. Similarly, published data have also implicated T helper type 2 (Th2)-derived cytokines, particularly IL-13, released during SLE flare due to dysregulated T-cells as one of the possible causes for podocytopathy [25]. Abnormal release of IL-13 from T-cells and cross-talk between renal dendritic cells and T helper cells can directly damage podocytes [26]. In addition, it has been shown that IL-13 induces CD80 expression in podocytes, resulting in foot process effacement and proteinuria [27]. In our patient, the serum level of IL-13 was not measured. Il-13 also is an important modulator of monocyte function; previous studies have shown that a monocyte factor is the mediator for increased glomerular permeability and proteinuria in MCD [28]. Podocytes are also known to express receptors for IL-4, IL-10 and IL-13, the presence of which during SLE flare can disrupt podocyte function [29]. IL-10 and IL-13 have an important role in Th2 cell differentiation and autoantibody production in SLE; hence T-cell abnormalities in both disorders may be the main unifying pathogenic mechanism in the development of MCD in SLE [30]. However, the role of IL-13 in LP needs to be studied as no published studies so far have looked at the relationship between the expression of IL-13 and induction of LP. Whether podocyte gene mutations also contribute to the pathogenesis will need to be studied. In our patient, nephrotic syndrome/FSGS gene panel were not sent. Additionally, several LP cases have been linked to nonsteroidal anti-inflammatory drug which are commonly used in arthralgia/arthritis associated with SLE [31].

Regarding T-cell dysfunction, a variety of observations are consistent with the hypothesis that cell-mediated immunity is a major pathogenic factor in both MCD and SLE [32]. In particular, the immature and relatively undifferentiated T-cells (CD34^+^) rather than the mature T-cells (CD34^−^) have been implicated in the pathogenesis of MCD, as proved in an animal study when proteinuria and foot process effacement were observed in mice after the engraftment of CD34^+^ but not CD34^−^ cells harvested from patients with glucocorticoid-sensitive MCD [33]. Glucocorticoids and cyclophosphamide, which modify cell-mediated responses, have proven benefit in the treatment of both MCD and SLE. Additionally, in children with glucocorticoid-sensitive NS, relapses were associated with a decrease in T regulatory cells [34]. B-cells also might play a significant role in the pathogenesis of MCD. Several studies have demonstrated favorable effect of rituximab, a chimeric monoclonal antibody that depletes the CD20^+^ B-cells, in inducing remission of NS. This suggests a probable glomerular permeability factor produced by B-cells through pathways regulated or stimulated by B-cells [35]. This evidence suggests roles of B-cells in addition to T-cells in the pathogenesis of MCD. In SLE, one of the typical characteristics is the formation of immune complexes with autoantibodies produced by B-cells that target various autoantigens, thus indicating the pivotal role of B-cells in the pathogenesis of SLE. Increasing evidence has shown abnormal expression of B-cells in the peripheral blood of SLE patients. Moreover, numerous studies have shown that B-cells in SLE patients are abnormally activated, as well as aberrantly differentiated, and are involved in the inflammatory cytokine milieu, abnormal transcription factor activity, and signaling pathways [36].

The patient described in this report had normal renal function and absence of proteinuria since the diagnosis at the age of 6 years, until the onset of NS at the age of 13 years. This patient had multiple risk factors for developing LN which include young age at initial presentation of SLE, AA race, positive family history of autoimmune diseases, long-standing hypertension, and history of Triple X syndrome with associated early puberty that results in exposure to estrogens at young age [5]. Estrogens are known modulators of immune system function, influence cytokine production and are involved in the lupus disease process as well [37,38]. It has been shown that the SLE is caused by impaired T-cell DNA methylation [39]. Demethylation of CD40LG, a B cell costimulatory molecule encoded on the inactive X chromosome, may contribute to the predilection of SLE in women. In patients with Triple X syndrome, each additional X chromosome, which is normally inactivated by methylation, could exert an increased incidence of SLE if methylation is reversed leading to a global reduction in methylation in activated T-cells of SLE patients [40]. Although there is enough data to support the increased incidences of lupus and Sjögren’s syndrome in patients with Triple X syndrome [40], it is unknown whether there is any link between Triple X and LP. Despite the above-mentioned risk factors, she did not have LN until seven years post SLE diagnosis when she presented with MCD. Hence, the reason for the delayed manifestation of MCD is unclear. It is possible that over time, the T and B cell dysfunction worsens and/or circulating immune factors increase which subsequently leads to podocytopathy. This needs to be studied further in future studies.

With regards to the differences in incidences of LP between adults and children, the authors were unable to find any comparative studies. However, there are some studies which have examined the incidences of lupus podocytopathy separately among adults and children. In the study by Hu et al. [21] who examined adult cases of SLE, 50 out of 3750 (1.33%) renal biopsies met the definition of lupus podocytopathy. In the retrospective pediatric study by Abdelnabi et al., the incidence of lupus podocytopathy was found to be as high as 8.1% [23].

The biologic agents have been implicated in the development of NS. Koya et al. described a 43-year-old man with resistant psoriasis who developed new onset MCD three months after initiation of etanercept, which resolved upon discontinuation of etanercept [41]). Additionally, biologics-induced autoimmune renal disorders have been described with etanercept, adalimumab, infliximab and others [42]. Direct effect of biologic agents on cytokine production and lymphocyte functions, and an increase in viral or bacterial infections in the setting of biologic treatment might promote autoimmune reactions via molecular mimicry, bystander activation or epitope spreading [42]. However, there also are reports describing the beneficial effect of biologics, including TNF α inhibitors, in the treatment of nephrotic syndrome [43]. TNFα causes down-regulation of nephrin, reorganizes the actin cytoskeleton, and causes dedifferentiation and podocyte proliferation [44]. Hence, resolution of MCD with the usage of steroid and while continuing etanercept makes etanercept induced MCD little less likely but will need to be studied further in future studies.

There is no evidence-based recommendation on how to treat patients with LP. However, it is reasonable to treat the NS with immunosuppression given likely role of circulating factors and/or T or B cell dysfunction. Steroids may be used as a first-line treatment for LP along with additional immunosuppressive agents, if needed. Multiple studies have shown that patients with a LP respond well to a short course of high-dose glucocorticoids [21], similar to patients with isolated idiopathic MCD as also shown in this report. Although most patients with LP respond to glucocorticoid therapy, there is a high incidence of recurrence, and the relapse rate could reach up to 90% on maintenance treatment with glucocorticoid alone [45]. Thus, glucocorticoid along with other immunosuppressive agents such as cyclophosphamide, tacrolimus or mycophenolate mofetil could significantly decrease the relapse rate, and KDIGO recommendations for the treatment of the initial episode and relapse of steroid sensitive nephrotic syndrome may be used as a guide for the treatment of LP.

### Limitations

Limitations of our report include absence of podocyte gene mutation testing, serum cytokines levels such as IL-13, and long-term follow-up. Genetic testing of the podocyte genes provide valuable information on the likelihood of steroid/immunosuppression responsiveness and risk of recurrence of NS post kidney transplantation should the NS progress to ESRD. Longer term follow-up is important to assess the risk of relapse and subsequent clinical outcome in these patients with LP.

## 4. Conclusions

This report highlights LP as another presentation of LN which is not included in the usual classification of LN. It also demonstrates MCD as a delayed renal manifestation of lupus in patients without renal involvement at the time of the diagnosis of SLE. LP represents a unique form of LN in patients with SLE. Current evidence suggests that LP is related to the SLE disease process itself rather than a mere coincidence. Hence, a renal histology is important for accurate diagnosis and proper management of NS in patients with SLE.
